# Prediction Nomogram for Postoperative 30-Day Mortality in Acute Type A Aortic Dissection Patients Receiving Total Aortic Arch Replacement With Frozen Elephant Trunk Technique

**DOI:** 10.3389/fcvm.2022.905908

**Published:** 2022-06-10

**Authors:** Hongyuan Lin, Yi Chang, Hongwei Guo, Xiangyang Qian, Xiaogang Sun, Cuntao Yu

**Affiliations:** Cardiac Surgery Centre, Fuwai Hospital, Chinese Academy of Medical Sciences and Peking Union Medical College, Beijing, China

**Keywords:** nomogram, prediction model, aortic dissection (AD), surgery, FET, machine learning (ML)

## Abstract

**Objective:**

To develop and validate a nomogram model to predict postoperative 30-day mortality in acute type A aortic dissection patients receiving total aortic arch replacement with frozen elephant trunk technique.

**Method:**

Clinical data on 1,156 consecutive acute type A aortic dissection patients who got total aortic arch replacement using the frozen elephant trunk technique was collected from January 2010 to December 2020. These patients were divided into training and testing cohorts at random with a ratio of 7:3. To predict postoperative 30-day mortality, a nomogram was established in the training set using the logistic regression model. The novel nomogram was then validated in the testing set. The nomogram's calibration and discrimination were evaluated. In addition, we created four machine learning prediction models in the training set. In terms of calibration and discrimination, the nomogram was compared to these machine learning models in testing set.

**Results:**

Left ventricular end-diastolic diameter <45 mm, estimated glomerular filtration rate <50 ml/min/1.73 m^2^, persistent abdominal pain, radiological celiac trunk malperfusion, concomitant coronary artery bypass grafting and cardiopulmonary bypass time >4 h were independent predictors of the 30-day mortality. The nomogram based on these 6 predictors manifested satisfying calibration and discrimination. In testing set, the nomogram outperformed the other 4 machine learning models.

**Conclusion:**

The novel nomogram is a simple and effective tool to predict 30-day mortality rate for acute type A aortic dissection patients undergoing total aortic arch replacement with frozen elephant trunk technique.

## Introduction

Acute type A aortic dissection (ATAAD) is a life-threatening disorder (1). The most effective treatment for the disease is emergent surgical repair (2). The surgical strategies for ATAAD lesions are varied due to the complicated and various nature of the lesions (3). Unlike western countries, in China, total aortic arch replacement (TAR) utilizing the frozen elephant trunk (FET) technique has become the most popular surgical procedure for ATAAD (4). As a complex procedure with higher risk, the outcome of TAR+FET is heavily influenced by the patient's preoperative status as well as the surgical components. Preoperative risk evaluation is of great importance in clinical practice and one of the most effective approaches is to use a prediction model. Although there are some prediction models for other heart procedures (5, 6), there are currently few models for TAR+FET surgery. Czerny et al. (7) established a model (GERAADA) for predicting the mortality of various ATAAD operations. GERAADA is, to our knowledge, the first multicenter data-based mortality risk prediction model for ATAAD surgery. It is a milestone in the risk evaluation of aortic dissection. However, a considerable percentage of GERAADA's training cohort had isolated ascending aortic (36.7%) or hemiarch (47.5%) replacement. Only 16% of the patients (*n* = 394) in their study had TAR surgery with or without FET. The aim of our research was to establish and evaluate a simple nomogram for predicting 30-day mortality in patients with ATAAD who underwent TAR+FET surgery.

## Methods

This study had been approved by institutional review board of Fuwai hospital, Peking union medical college and Chinese academy of medical sciences. Institutional Review Board (IRB) approval number: 2020-1402. Date: 2020-11-24.

### Patients

**Figure 1:**
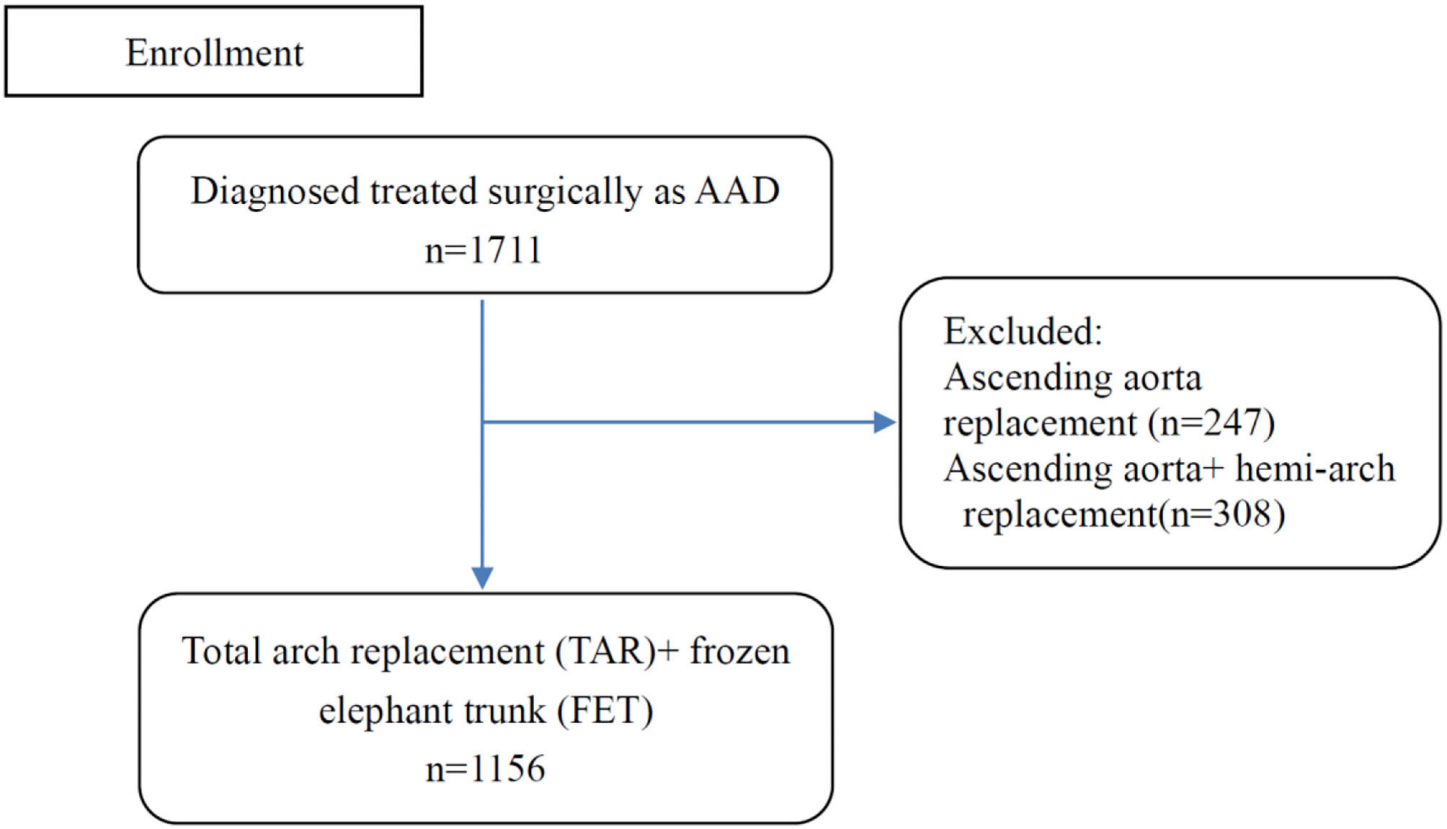
Flow chart of patient enrollment.

All patients (*n* = 1,711) who were diagnosed with ATAAD and underwent surgical repair at Fuwai Hospital were enrolled from January 2010 to December 2020. Total 247 patients with isolated ascending aorta replacement and 308 patients with ascending aorta with hemi-arch replacement were excluded from the final study, remaining 1,156 individuals with TAR+FET (Figure 1). The acute phase of aortic dissection was defined as the time between onset and surgery, which was less than 2 weeks. In our institution, the indications for ATAAD patients to receive TAR+FET were as follow: (1) the dissection involved the distal aortic arch and/or descending aorta; (2) Aneurysm formation in the distal aortic arch and/or descending aorta (transverse diameter >40 mm). We collected demographic data, preoperative risk factors and important intraoperative information of all patients for analysis.

### Preoperative Evaluation

All patients underwent preoperative radiological examinations such as aortic computed tomograph angiography (CTA, scanning from the level of the supra aortic vessels to bilateral femoral arteries), transthoracic echocardiography (TTE) and coronary CTA.

### Surgical Technique

After cardiopulmonary bypass (CPB) established, ascending aorta was clamped and cardioplegia was transmitted to coronary orifice directly. Aortic root repair was done during cooling phase. Circulatory arrest was instituted, if the nasopharyngeal temperature reached target temperature (usually 18–22°C). The supra-arch arteries were clamped and the aortic arch was open. Selective cerebral perfusion (SCP) was started through the right axillary artery. Aortic arch was resected between the origin of the left subclavian artery and the left carotid artery and supra-arch arteries were resected at their initial part. The FET was deployed into the true lumen of distal aorta. The distal aorta incorporating the stent graft was sewn to the distal end of a 4-branch prosthetic graft using distal first technique. After completion of distal anastomosis, the graft was cross-clamped, and antegrade systemic perfusion was achieved through a side branch.

The sequence of supra-arch arteries anastomosing to prosthetic graft branches was carried out from left common carotid artery, left subclavian artery, and innominate artery. Sometimes innominate artery was anastomosed after proximal aortic stump anastomosis was completed. Usually after the anastomosis of left common carotid artery was accomplished, SCP was discontinued, CPB gradually resumed to normal flow, and rewarming started.

### Definition of Parameters

To identify the parameters that would influence the progress and outcome of ATAAD, we defined some institution-specific terms to describe them in **Table 2**.

### Statistical Analysis

We followed the Transparent Reporting of a Multivariable Prediction Model for Individual Prognosis or Diagnosis (TRIPOD) statement for reporting the development and validation of the prediction model (8). With a ratio of 7:3, all the patients were randomly divided into training set (*n* = 806) and testing set (*n* = 350). Categorical variables were presented as frequencies (percentages %) and were compared between groups with the chi-squared test. Continuous variables were presented as mean ± standard deviation (SD), and were compared with the two-sample *t* test or the Wilcoxon rank sum test as appropriate. According to restricted cubic spline analysis, we also converted some continuous variables into binary variables (Supplementary Figures 1–3). *P* value of > 0.05 was considered statistically significant.

**Figure 2:**
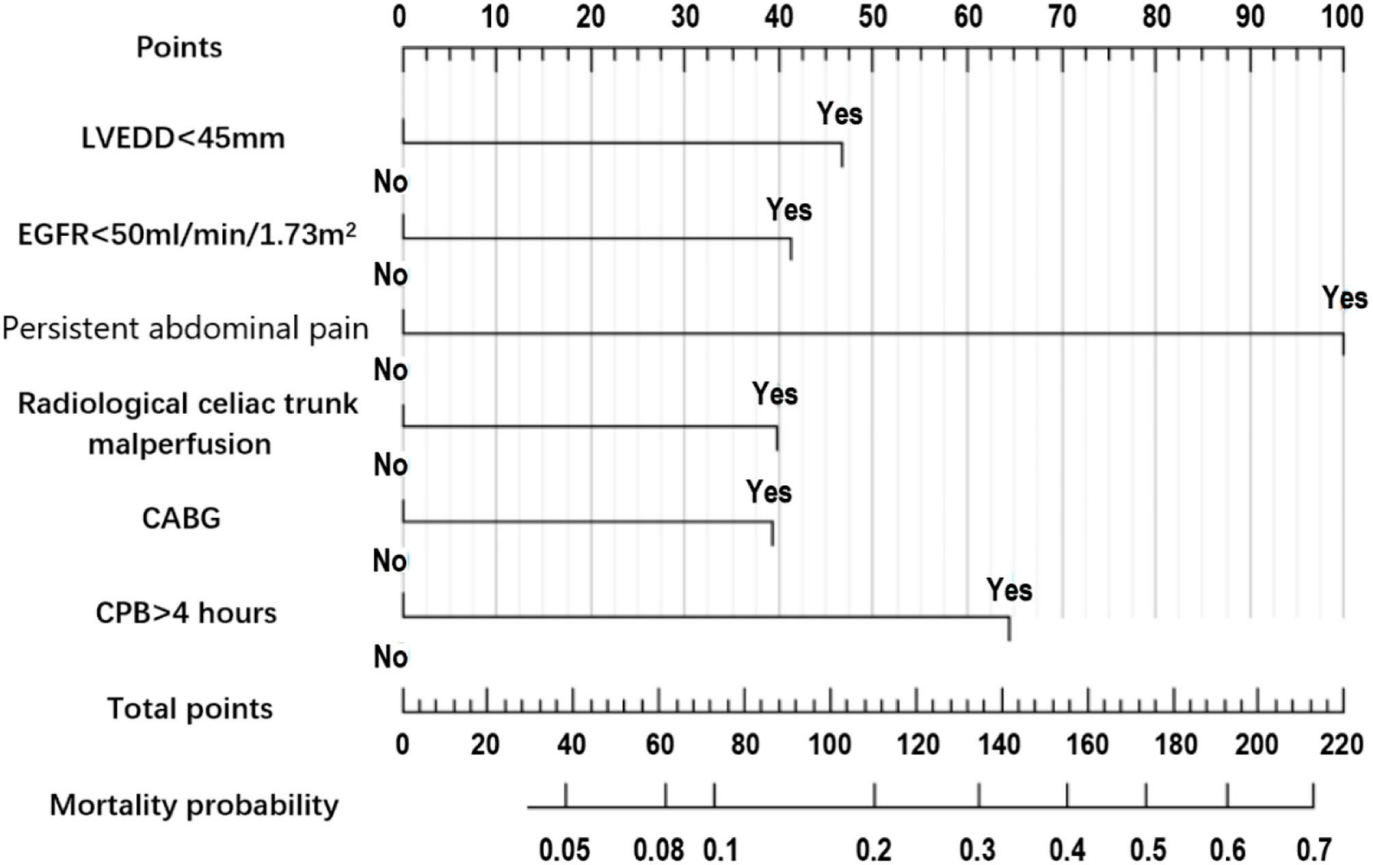
Nomogram prediction model of 30-day mortality in acute aortic dissection patients undergoing total arch replacement surgery.

In training set, all the possible risk factors were screened by univariate analyses and a multivariate logistic regression was performed using “enter” method to construct the initial model. Next, to construct the final model, we performed the variable selection process by repeated multivariate logistic regression analyses using “stepwise both direction” method. The calculation of Akaike weights confirmed the importance of the final model parameters. Subsequently, we constructed a nomogram based on the last logistic regression model (Figure 2). Additionally, in the training set, using algorithms of naive Bayesian (NB) classification, support vector machine (SVM), random forest (RF) and extreme gradient boosting (XGB), we also screened all variables shown in Table 1 and constructed 4 machine learning (ML) models.

**Table 1:** Patient demographics and clinical features.

**Variables**	**Training (*n* = 806)**	**Testing (*n* = 350)**	***p*-value**
**Patient-related**			
Age, mean ± SD	46.95 ± 9.92	46.90 ± 10.27	0.939
Female, *n*(%)	160 (19.9)	73 (20.9)	0.755
BMI, mean ± SD	26.36 ± 4.21	26.12 ± 5.10	0.4
Marfan syndrome, *n*(%)	60 (7.4)	30 (8.6)	0.591
Diabetes mellitus, *n*(%)	23 (2.9)	4 (1.1)	0.119
Previous stroke, *n*(%)	33 (4.1)	13 (3.7)	0.889
Chronic kidney disease, *n*(%)	5 (0.6)	4 (1.1)	0.572
COPD, *n*(%)	2 (0.2)	2 (0.6)	0.753
ALT (unit/L), mean ± SD	30.87 ± 36.86	40.30 ± 12.78	0.224
EGFR (ml/min/1.73m^2^), mean ± SD	101.35 ± 38.21	99.48 ± 34.64	0.432
EGFR <50 ml/min/1.73m^2^, *n*(%)	45 (5.6)	19 (5.4)	1
NYHA III or IV, n(%)	166 (20.6)	85 (24.3)	0.187
Hypertension, n(%)	520 (64.5)	224 (64.0)	0.919
Coronary artery disease, *n*(%)	58 (7.2)	23 (6.6)	0.797
Atrial fibrillation, *n*(%)	8 (1.0)	3 (0.9)	1
Previous cardiovascular surgery, *n*(%)	20 (2.5)	8 (2.3)	1
Previous TEVAR, *n*(%)	12 (1.5)	3 (0.9)	0.556
Instable hemodynamics, *n*(%)	35 (4.3)	23 (6.6)	0.147
**TTE-related**			
LVEDD <45 mm, *n*(%)	90 (11.2)	47 (13.4)	0.32
Ejection fraction, *n*(%)	60.35 (4.31)	60.04 (4.63)	0.276
Severe aortic regurgitation, *n*(%)	81 (10.0)	50 (14.3)	0.051
Hydropericardium, *n*(%)	11 (1.4)	5 (1.4)	1
**Dissection-related**			
Severely compressed true lumenin descending aorta, *n*(%)	78 (9.7)	29 (8.3)	0.522
Persistent abdominal pain, *n*(%)	9 (1.1)	5 (1.4)	0.878
Lower limb ischemia, *n*(%)	54 (6.7)	18 (5.1)	0.382
Clinical coronary ostium involved, *n*(%)	116 (14.4)	50 (14.3)	1
Carotid ostium involved*, n*(%)	478 (59.3)	205 (58.6)	0.867
Radiological Celiac trunk malperfusion, *n*(%)	74 (9.2)	37 (10.6)	0.53
Radiological superior mesenteric artery malperfusion, *n*(%)	56 (6.9)	32 (9.1)	0.241
Renal malperfusion, *n*(%)	40 (5.0)	28 (8.0)	0.06
Radiological iliac-femoralmalperfusion, n(%)	82 (10.2)	37 (10.6)	0.921
**Operation-related**			
Concomitant with aortic root surgery, *n*(%)	216 (26.8)	108 (30.9)	0.18
CABG, *n*(%)	104 (12.9)	42 (12.0)	0.743
Salvage CABG, *n*(%)	14 (1.7)	8 (2.3)	0.694
Carotid bypass, *n*(%)	30 (3.7)	5 (1.4)	0.057
CPB time > 4 h, *n*(%)	146 (18.1)	58 (16.6)	0.584
DHCA temperature (degree centigrade), mean ± SD	22.04 ± 3.58	21.74 ± 3.39	0.179
DHCA time (minutes), mean ± SD	18.57 ± 6.90	19.37 ± 6.59	0.067
**Outcome**			
30-day postoperative deaths, *n*(%)	43 (5.3)	27 (7.7)	0.154

*SD, standard deviation; BMI, body mass index; COPD, chronic obstructive pulmonary disease; ALT, Alanine Aminotransferase; EGFR, estimated glomerular filtration rate; NYHA, New York heart association; TEVAR, thoracic endovascular aortic repair; TTE, transthoracic echocardiography; LVEDD, left ventricular end-diastolic diameter; CTA, computed tomographic angiography; CABG, coronary artery bypass grafting; CPB, cardiopulmonary bypass; DHCA, deep hypothermia and circulatory arrest*.

The calibration plot and Brier score were used to evaluate the calibration of the nomogram. The receiver operating characteristic (ROC) curve and the area under the receiver operating characteristic curve (AUC) were used to evaluate the discrimination of the model. In the testing set, the nomogram was compared to the other four ML models in terms of Brier scores for calibration and AUCs for discrimination. R software version 4.0.2 was used for statistical analysis. Graph pad Prism for Windows version 6.0 was used to create graphs.

## Results

### Demographics and Predictor Candidates of Training and Testing Sets

Figure 1 depicted a flow chart of patient enrollment. Table 1 presented a comparison of demographics and predictor candidates between the training and testing sets. With respect to these risk factors, there were no differences between the two groups. Table 2 contained the definitions or explanations for all of the variables.

**Table 2:** Definitions of risk factors.

**Variables**	**Definitions**
Age	-
Female	-
BMI	Body mass index
Marfan syndrome	Documented past history or fulfilled the revised Ghent criteria
Diabetes mellitus	Documented past history or fulfilled the criteria of WHO 1999
Previous stroke	Documented past history
Chronic kidney disease	Documented past history or fulfilled the criteria of KDIGO 2012
COPD	Long-term use of bronchodilators or steroids for lung disease
ALT	-
EGFR	Estimated by the Modification of Diet in Renal Disease (MDRD) equation
NYHA III or IV	NYHA classification
Hypertension	Documented past history or SBP>140 mmHg and/or DBP > 90 mmHg
Coronary artery disease	Documented past history
Atrial fibrillation	Documented past history
Previous cardiovascular surgery	1 or more previous major cardiac operation involving opening the pericardium
Previous TEVAR	Documented past history
Instable hemodynamics	Need for catecholamines at referral
LVEDD	-
Ejection fraction	-
Severe aortic regurgitation	-
Hydropericardium	Massive pericardiac fluid
Severely compressed true lumenin descending aorta	Revealed by CTA
Persistent abdominal pain	Persistent severe abdominal pain associated with aortic dissection
Lower limb ischemia	Symptoms or signs of lower limb ischemia, such as pain, numbness, or weak pulse of dorsal foot artery etc.
Clinical coronary ostium involved	The coronary ostium lesion confirmed in operation
Carotid ostium involved	Carotid ostium lesion confirmed in operation
Radiological celiac trunk malperfusion	Celiac trunk malperfusion revealed by CTA
Radiological superior mesenteric artery malperfusion	Superior mesenteric artery malperfusion revealed by CTA
Renal malperfusion	Unilateral or bilateral renal malperfusion revealed by CTA, regardless of renal function
Radiological iliac-femoral malperfusion	Unilateral or bilateral iliac-femoral malperfusion revealed by CTA
Concomitant with aortic root surgery	Combined with bentall or valve sparing root replacement surgery
CABG	Combined with CABG surgery
Salvage CABG	The CABG was not planned preoperatively, and was performed because a post-operative hemodynamic instability (such as malignant arrhythmia or failing to wean from cardiopulmonary bypass) occurred with suspicion of myocardial ischemia
Carotid bypass	Combined with uni- or bilateral carotid bypass surgery.
CPB time > 4 h	-
DHCA temperature	Nasopharyngeal temperature
DHCA time	-
30-day postoperative deaths	All-cause mortality

*BMI, body mass index; WHO, world health organization; KDIGO, kidney disease, improving global outcomes; COPD, chronic obstructive pulmonary disease; ALT, Alanine Aminotransferase; EGFR, estimated glomerular filtration rate; NYHA, New York heart association; SBP, systolic blood pressure; DBP, diastolic blood pressure; TEVAR, thoracic endovascular aortic repair; TTE, transthoracic echocardiography; LVEDD, left ventricular end-diastolic diameter; CTA, computed tomographic angiography; CABG, coronary artery bypass grafting; CPB, cardiopulmonary bypass; DHCA, deep hypothermia and circulatory arrest*.

Postoperative outcomes 90 patients (6.1%) died within 30 days of surgery in the entire cohort (*n* = 1,156). Other major postoperative complications included 135 patients (11.7%) who received continuous renal replacement therapy (CRRT), 68 patients (5.9%) who had a stroke, 122 patients (10.6%) who had a prolonged ventilation time (>96 h), and 201 patients (17.4%) who were in the intensive care unit (ICU) for more than 7 days.

### Univariate and Multivariate Analyses

The summary of univariate analyses was shown in Supplementary Table 1. The results of the multivariate analysis for the initial model before the variable selection were shown in Supplementary Table 2. The results of the multivariate analysis for the final model after the variable selection were shown in Table 3.The independent predictors selected to establish the final model were: Left ventricular end-diastolic diameter (LVEDD) <45 mm, estimated glomerular filtration rate (eGFR) <50 ml/min/1.73m^2^, persistent abdominal pain, radiological celiac trunk malperfusion, concomitant coronary artery bypass grafting (CABG) and cardiopulmonary bypass (CPB) time > 4 h.

**Table 3:** Multivariable analysis of perioperative parameters.

**Variables**	**Coefficients**	**SE**	**Wald**	***p-*value**
LVEDD <45 mm	1.0128	0.314	3.225	0.001**
EGFR <50 ml/min/1.73m^2^	0.8983	0.4127	2.177	0.03*
Persistent abdominal pain	2.174	0.6318	3.441	<0.001***
Previous stroke	0.6512	0.5279	1.234	0.217
Clinical coronary ostium lesion	0.3637	0.5054	0.72	0.472
Radiological celiac trunk malperfusion	0.8631	0.3553	2.429	0.015*
CABG	0.8535	0.3122	2.734	0.006**
CPB time > 4 h	1.4003	0.2897	4.833	<0.001***
Age	0.014	0.0137	1.022	0.307
Intercept	−3.7806	0.2173	−17.4	

SE, standard error; LVEDD, left ventricular end-diastolic diameter; EGFR, estimated glomerular filtration rate; CABG, coronary artery bypass grafting; CPB, cardiopulmonary bypass. *0.01 ≤ p < 0.05; **0.001 ≤ p < 0.05; ***p < 0.001.

### Model Development

**Figure 3:**
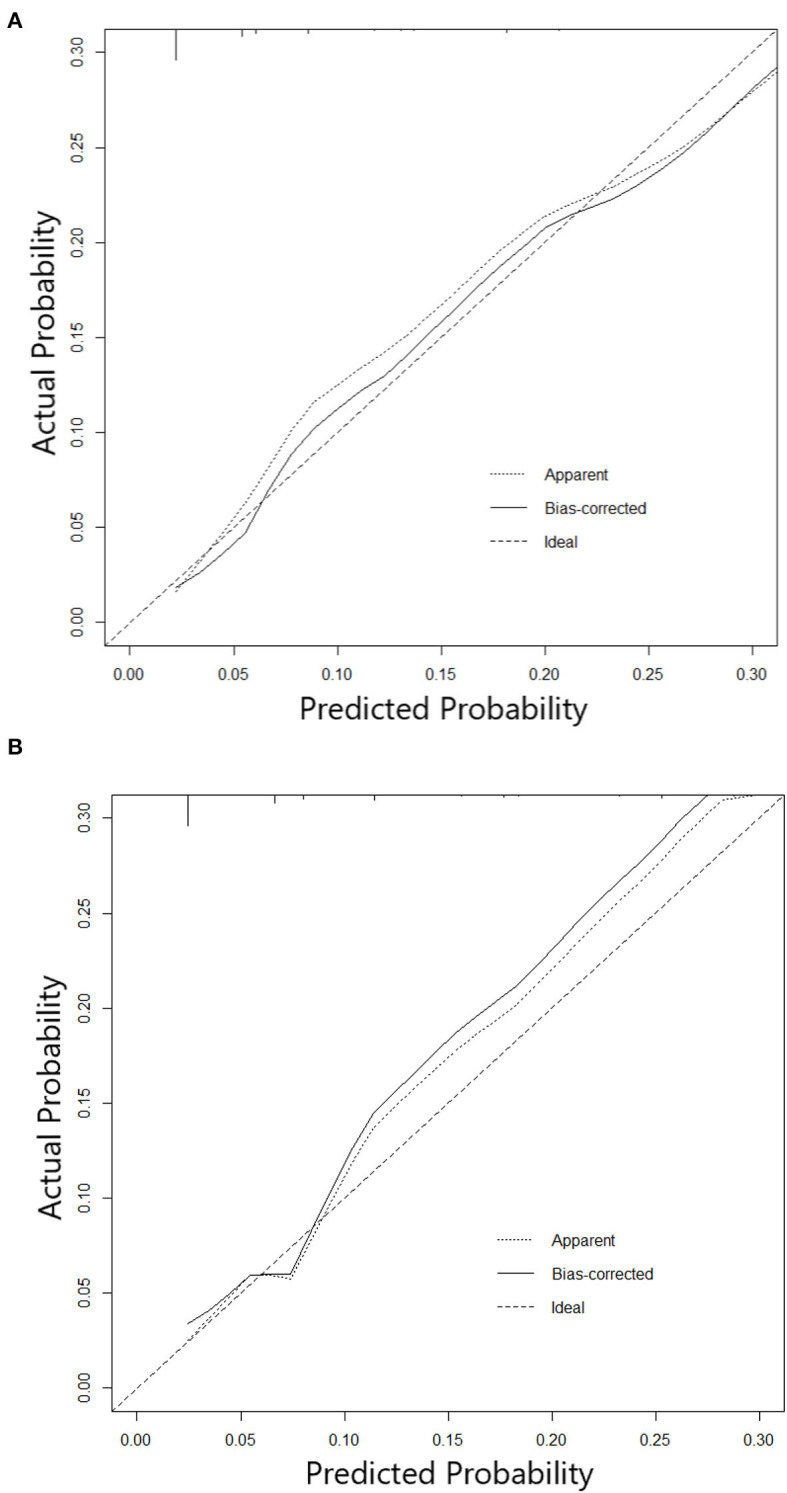
**(A)** Calibration curve of Nomogram in training cohort. **(B)** Calibration curve of Nomogram in testing cohort.

**Figure 4:**
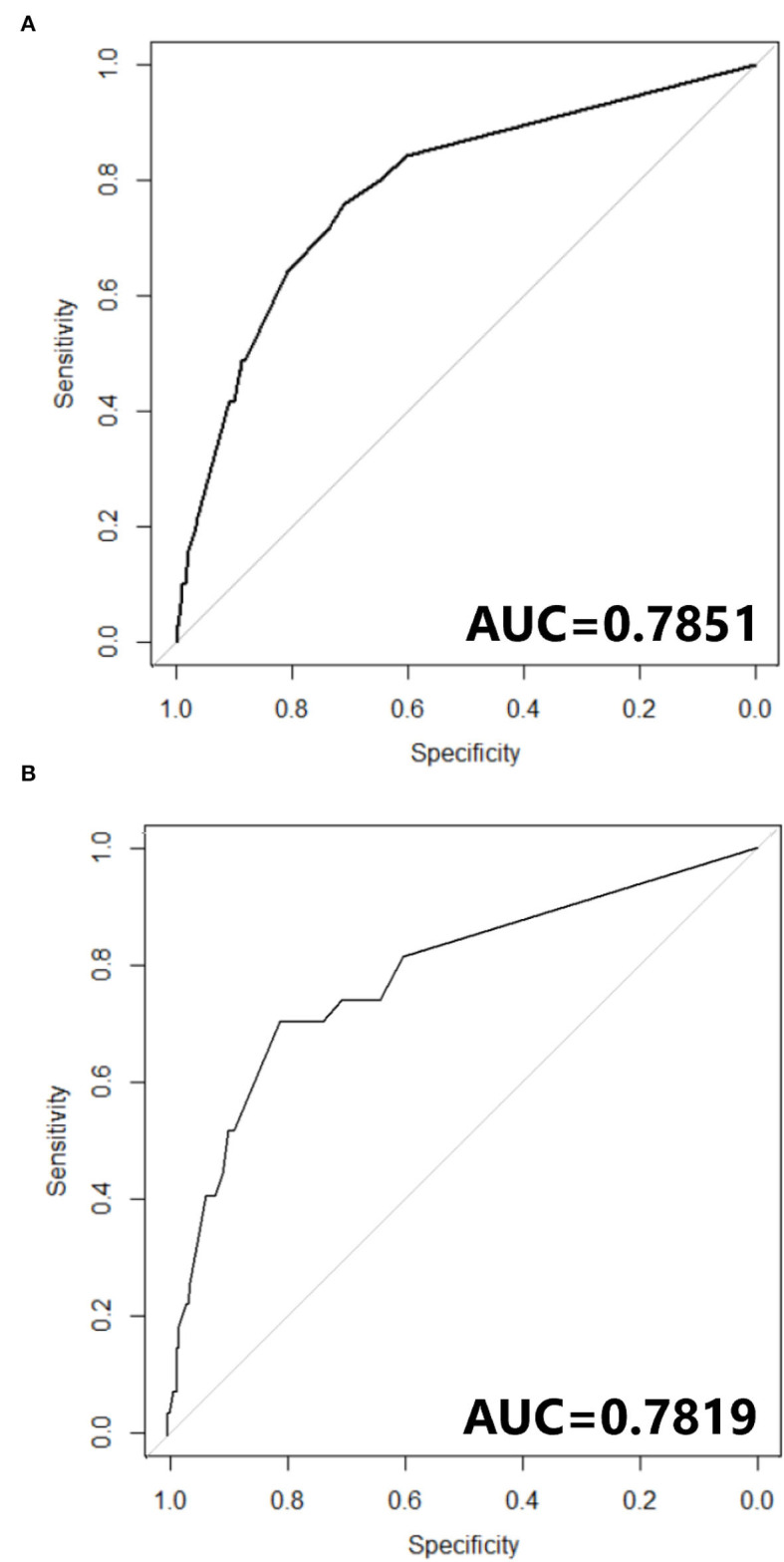
**(A)** ROC curve of Nomogram in training cohort. **(B)** ROC curve of Nomogram in testing cohort.

The nomogram constructed according to the final logistic regression model was shown in Figure 2. In training set, the calibration plot was displayed in Figure 3A, the Brier score was 0.0523 and the ROC curve was showed in Figure 4A, with an AUC (c-index) of 0.7851.

### Model Validation and Compared With Other Machine Learning Algorithms

**Figure 5:**
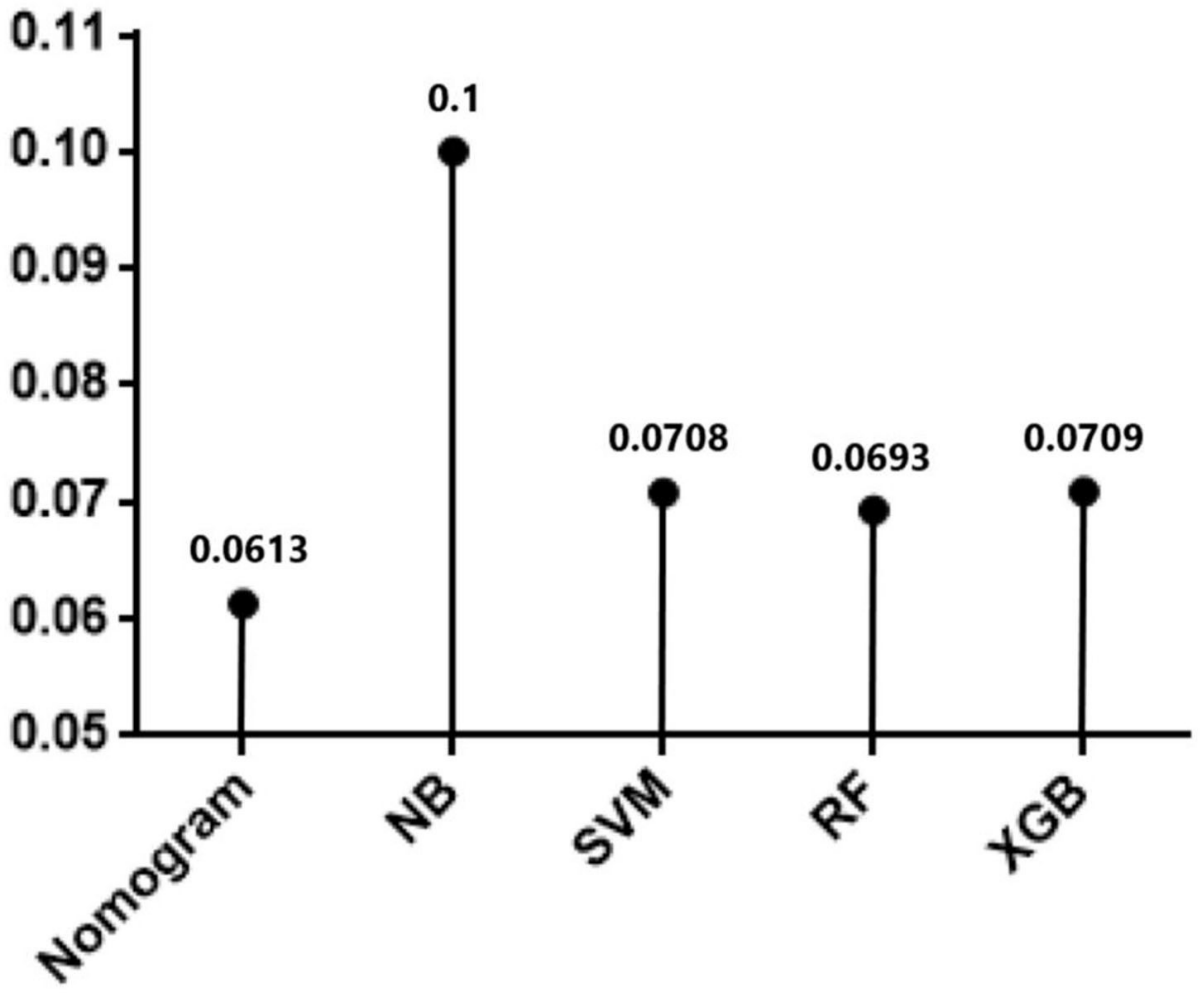
Brier scores of Nomogram and the other 4 machine learning models in testing cohort.

**Figure 6:**
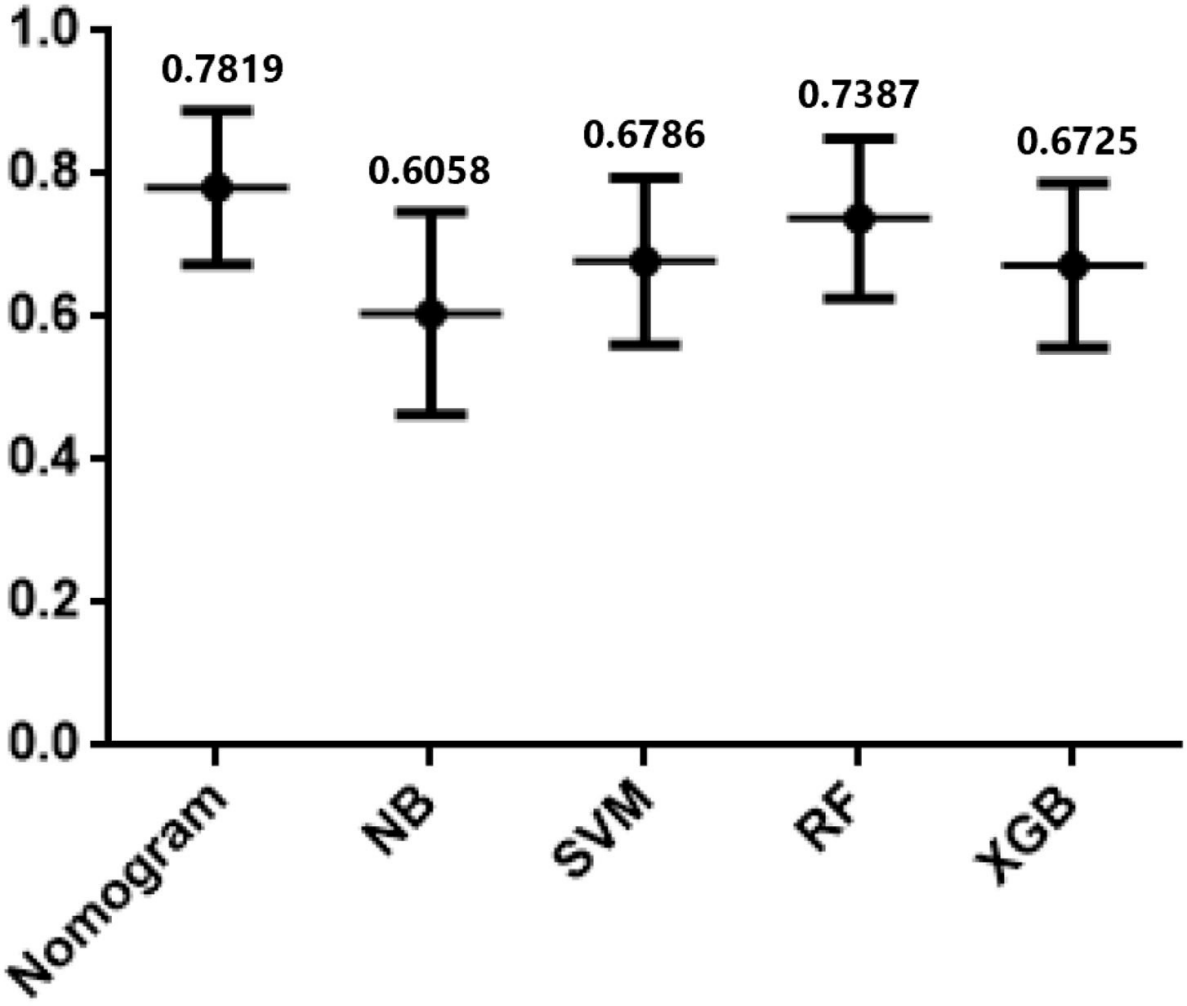
AUCs of Nomogram and the other 4 machine learning models in testing cohort.

In testing set, the calibration plot of the nomogram was showed in Figure 3B, the Brier score was 0.0613. The ROC curve was showed in Figure 4B, with a c-index of 0.7819. Figure 5 demonstrated Brier scores of the nomogram and other ML models. We could find a lower Brier score of the nomogram than the other 4 ML models, which suggested superior calibration of the nomogram. Figure 6 presented AUC values with 95% confidence intervals (95% *CIs*) of the nomogram and ML models. We could observe a larger AUC of the nomogram than the other 4 ML models, which suggested better discrimination of the nomogram.

## Discussion

The nomogram is a simple, pictorial, and effective tool for predicting the 30-day mortality rate in ATAAD patients who do have TAR+FET surgery. This nomogram only included 6 easily accessible predictors, less than the GERAADA score (7) (11 predictors) and Euroscore II (5) (more than 20 predictors). As the nomogram is convenient and user-friendly for clinical evaluation, we recommend that this bedside tool be used widely in clinical practice.

The surgical management of extensive dissection affecting the aortic arch and descending aorta is difficult. TAR+FET combine the advantages of open surgical techniques and endovascular intervention, allowing simultaneous aortic arch repair and stabilization of the descending aorta. As a result of exactly sealed anastomosis of FET and proximal graft, TAR+FET can eliminate the risk of type I endoleak and retrograde aortic dissection. Meanwhile, FET could also benefit the thrombosis of false lumen, enable positive aortic remodeling (9). Additionally, TAR+FET could not only simplify the second-phase operation of the thoraco-abdominal aorta after arch repair in some extensive aortic dissection cases, (10) but also establish a stable and durable proximal landing zone for TEVAR if necessary. Kreibich, M et al. conducted a study (11) (*n* = 66) showing that downstream TEVAR following the FET procedure was associated with excellent clinical outcomes. Furthermore, in those with connective tissue disorder, like marfan syndrome, TAR+FET can be safely performed with favorable long-term survival and freedom from reoperation (12, 13). Berger, T and Kreibich, M et al. reported their studies (14, 15) suggesting that use of FET could provide acceptable morbidity and mortality for reintervention in patients requiring staged operations due to extensive aortic disease (concomitant thoraco-abdominal aortic pathologies).

Tamura et al. (16) conducted a retrospective study on ATAAD and discovered that TAR+FET were much more commonly chosen for patients under the age of 50. In fact, ATAAD patients in China are substantially younger than those in Western countries. In this study, the mean age was 46.9 ± 10.0 years, whereas the average age was 61.3 ± 13.5 years in GERAADA study cohort (7) and 61.5 ± 14.6 years in the International Registry of Acute Aortic Dissection (IRAD) cohort (17). As the life expectancy is longer in Chinese patients, surgeons prefer the TAR+FET strategy for its more favorable long-term outcomes. Consequently, in China, TAR+FET has become a widespread standard surgical method for ATAAD involving the entire aortic arch and descending aorta (4).

Prediction models should be region-specific: models designed for Western populations may be less appropriate to Asian or Chinese populations (18). Wessler et al. (19) conducted a research suggesting that clinical prediction models may have variable performance across various databases, especially in many areas of Eastern Europe, Asia, Central America, South America, and Africa where much remains unknown. This is especially important considering the many regional differences in etiology, access to technologies, care systems, and guidelines. Nežić et al. (20) reported their validation study (*n* = 137) on GERAADA score revealing that the discrimination of GERAADA score was poor, with an AUC of 0.55, much lower than the EuroSCORE II score (AUC = 0.799). Therefore, developing a prediction model for TAR+FET in the Chinese population is of major clinical importance.

In this study, we discovered that persistent abdominal pain and radiological celiac trunk malperfusion were independent risk factors for postoperative mortality, particularly persistent abdominal pain, which had a greater predictive power. The findings confirmed that visceral malperfusion was an immediate life-threatening concern in ATAAD patients, with 70 to 100% postoperative death (21). This fact motivated ATAAD patients to undergo early visceral perfusion assessment and, for certain cases, endovascular reperfusion prior to surgical aortic repair.

Interestingly, we noted that preoperative LVEDD <45 mm revealed by preoperative TTE was also an independent predictor for mortality. As an indicator of cardiac preload, low LVEDD might lead to inadequate cardiac output or end-organ perfusion which is in associated with mortality or neurological events (22, 23). The results indicated that insufficient cardiac preload or hypotension should be avoided in preoperative volume therapy. In addition, the enlarged false lumen in some of the ATAAD cases could store a large amount of blood, further reducing the circulating blood volume, manifested by low LVEDD or hypotension.

In current study, the nomogram demonstrated superior predictive performance than other ML models; with a lower Brier score (Figure 5) and higher AUC (Figure 6). As we all know, machine learning algorithms are frequently applied to larger datasets. Although the sample size in this study is sufficient for TAR+FET, it is at bit small for ML algorithms, which may result in poor ML model performance. Although the ML algorithms adopted in prediction model development is a popular subject nowadays, we still need to focus a lot more on thorough and accurate clinical data gathering, which is the foundation of clinical research.

This nomogram is not a reference to accept or reject a certain treatment. For ATAAD, open surgical repair is often a life-saving solution and remains the standard of care for patients. The nomogram is only a bedside tool to help clinical practitioners make a rapid initial prognosis assessment for patients receiving TAR+FET. We hope this novel nomogram could quickly identify potential risk factors and provide the opportunity for timely and prompt intervention during the emergency course of treatment.

### Limitations

Due to the single center research, we only conducted internal validation. The nomogram's predictive ability needs to be evaluated by external validation using clinical data from other centers. Pending for further external validations; this model can only be applied to comparable cohorts since Chinese patient cohorts differ substantially from European or the US.

## Conclusion

The novel nomogram is a simple and effective tool to predict 30-day mortality rate for acute type A aortic dissection patients undergoing total aortic arch replacement with frozen elephant trunk technique.

## Data Availability Statement

The original contributions presented in the study are included in the article/Supplementary Materials, further inquiries can be directed to the corresponding author/s.

## Author Contributions

HL analyzed the data, collected the data, and was a major contributor in writing the manuscript. HG, XQ, XS, and CY were in charge of the surgery performing. YC was in charge of the study design, data collection, and manuscript modification. All authors read and approved the final manuscript.

## Funding

The current study and YC and HL were supported by CAMS Innovation Fund for Medical Sciences (CIFMS), 2020-I2M-C&T-B-064.

## Conflict of Interest

The authors declare that the research was conducted in the absence of any commercial or financial relationships that could be construed as a potential conflict of interest.

## Publisher's Note

All claims expressed in this article are solely those of the authors and do not necessarily represent those of their affiliated organizations, or those of the publisher, the editors and the reviewers. Any product that may be evaluated in this article, or claim that may be made by its manufacturer, is not guaranteed or endorsed by the publisher.
