# Supplementary material for: Prediction Nomogram for Postoperative 30-Day Mortality in Acute Type A Aortic Dissection Patients Receiving Total Aortic Arch Replacement With Frozen Elephant Trunk Technique
Source: Front Cardiovasc Med. 2022 Jun 10;9:905908. doi: 10.3389/fcvm.2022.905908 (PMC9226415; doi:10.3389/fcvm.2022.905908)
Supplement: Supplementary file 1 [file Table_1.docx]

| Variables | Pearson χ^2^ value | P value |
| --- | --- | --- |
| Female | 2.493 | 0.139 |
| NYHA III or IV | 1.719 | 0.221 |
| LVEDD<45mm | 9.7976 | 0.0017 |
| EGFR<50ml/min/1.73m^2^ | 6.2185 | 0.01264 |
| Persistent abdominal pain | 14.765 | 0.000 |
| Lower limb ischemia | 2.5673 | 0.109 |
| Hypertension | 2.278 | 0.139 |
| Coronary artery disease | 0.768 | 0.384 |
| Marfan syndrome | 0.563 | 0.613 |
| Previous cardiovascular surgery | 1.214 | 0.621 |
| Previous TEVAR | 0.132 | 0.519 |
| Diabetes mellitus | 1.208 | 0.625 |
| Previous stroke | 8.847 | 0.012 |
| Chronic kidney disease | 0.449 | 0.422 |
| Severe aortic regurgitation | 0.024 | 1 |
| Hydropericardium | 0.041 | 0.575 |
| Severely compressed true lumen in descending aorta | 0.043 | 0.8 |
| Clinical coronary ostium involved | 8.8251 | 0.002 |
| Carotid ostium involved | 0.590 | 0.430 |
| Radiological Celiac trunk malperfusion | 5.849 | 0.015 |
| Radiological superior mesenteric artery malperfusion | 3.190 | 0.091 |
| Renal malperfusion | 1.211 | 0.622 |
| Radiological iliac-femoral malperfusion | 0.207 | 0.628 |
| Concomitant with aortic root surgery | 1.102 | 0.339 |
| CABG | 29.614 | 0.000 |
| Salvage CABG | 8.173 | 0.004 |
| Carotid bypass | 0.117 | 0.669 |
| Instable hemodynamics | 0.073 | 0.739 |
| CPB time>4 hours | 46.79 | 0.000 |
| Continues variables | | |
| Age | T test | 0.047 |
| Weight | Wilcoxon test | 0.923 |
| Height | Wilcoxon test | 0.574 |
| BMI | Wilcoxon test | 0.568 |
| ALT | Wilcoxon test | 0.134 |
| DHCAtime | Wilcoxon test | 0.2109 |
| DHCAtemp | Wilcoxon test | 0.1251 |

Supplement table 1. Summary of univariate analysis
