# Supplementary material for: Prediction Nomogram for Postoperative 30-Day Mortality in Acute Type A Aortic Dissection Patients Receiving Total Aortic Arch Replacement With Frozen Elephant Trunk Technique
Source: Front Cardiovasc Med. 2022 Jun 10;9:905908. doi: 10.3389/fcvm.2022.905908 (PMC9226415; doi:10.3389/fcvm.2022.905908)
Supplement: Supplementary file 2 [file Table_2.docx]

Supplement table 2. Results of multivariable logistic regression for the initial model.

| Variables | coefficients | SE | Wald | P value |
| --- | --- | --- | --- | --- |
| LVEDD<45mm | 0.9416 | 0.3263 | 2.885 | 0.004** |
| EGFR<50ml/min/1.73m^2^ | 0.83526 | 0.4289 | 1.947 | 0.052 |
| Persistent abdominal pain | 2.33041 | 0.6316 | 3.690 | <0.001*** |
| Previous stroke | 0.6512 | 0.5279 | 1.234 | 0.217 |
| Clinical coronary ostium lesion | 0.3637 | 0.5054 | 0.72 | 0.472 |
| Radiological Celiac trunk malperfusion | 0.8431 | 0.3603 | 2.34 | 0.019* |
| CABG | 0.63184 | 0.35623 | 1.774 | 0.076 |
| CPB time>4 hours | 1.2904 | 0.3185 | 4.05 | <0.001*** |
| Age | 0.014 | 0.0137 | 1.022 | 0.307 |
| Intercept | -4.476 | 0.6928 | -6.461 |  |
