# Supplementary figures and images for: Prediction Nomogram for Postoperative 30-Day Mortality in Acute Type A Aortic Dissection Patients Receiving Total Aortic Arch Replacement With Frozen Elephant Trunk Technique
Source: Front Cardiovasc Med. 2022 Jun 10;9:905908. doi: 10.3389/fcvm.2022.905908 (PMC9226415; doi:10.3389/fcvm.2022.905908)

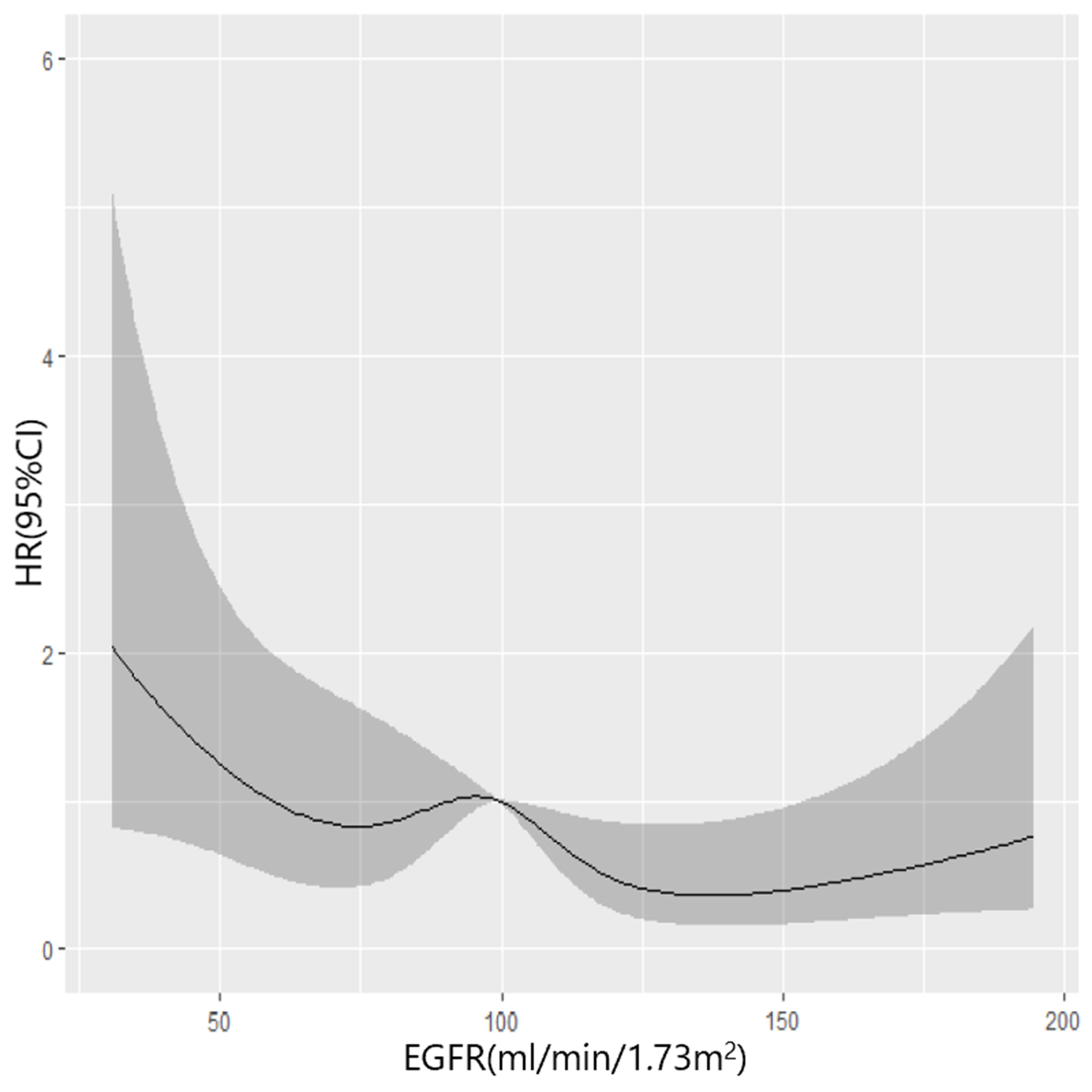

Supplement: Supplementary file 3 [file Image_1.TIFF]

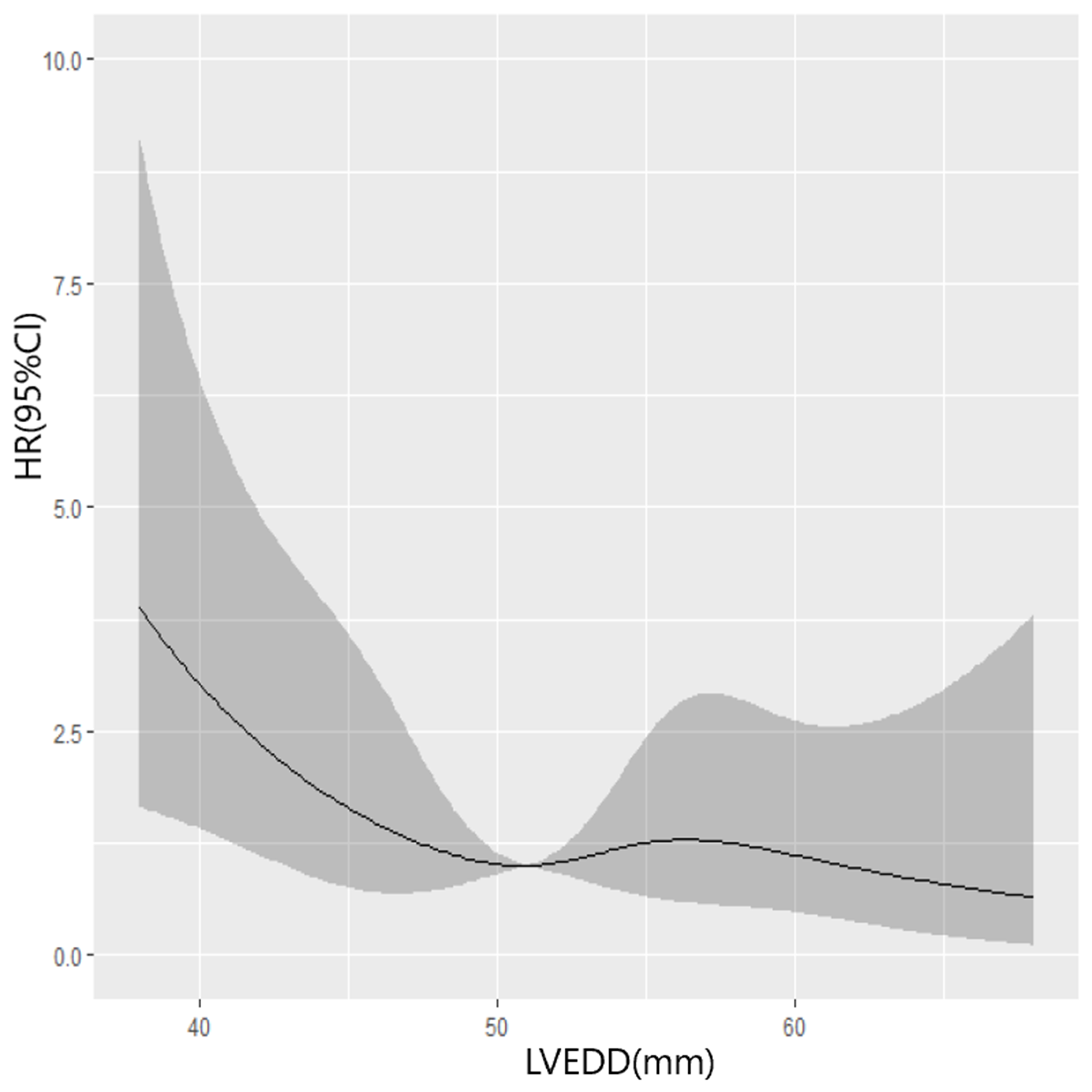

Supplement: Supplementary file 4 [file Image_2.TIFF]

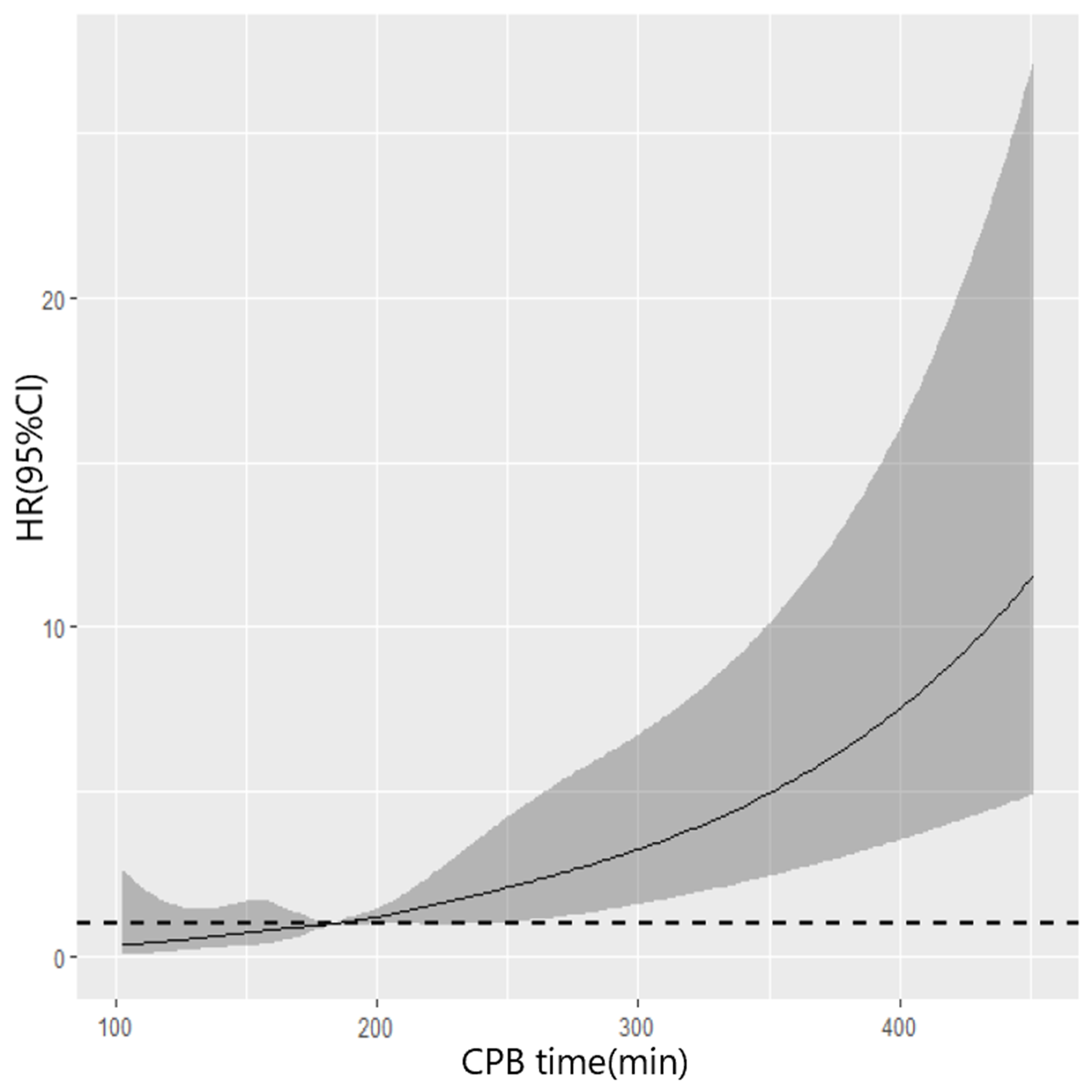

Supplement: Supplementary file 5 [file Image_3.TIFF]
